# Supplementary material for: Combinations of PCR and Isothermal Amplification Techniques Are Suitable for Fast and Sensitive Detection of SARS-CoV-2 Viral RNA
Source: Front Bioeng Biotechnol. 2020 Nov 4;8:604793. doi: 10.3389/fbioe.2020.604793 (PMC7672014; doi:10.3389/fbioe.2020.604793)

Supplementary Material

# Supplementary Table

**Supplementary Table S1.** Primer sequences used in polymerase chain reaction (PCR), polymerase chain displacement reaction (PCDR), loop-mediated isothermal amplification (LAMP), and PCR-LAMP assays.

| **Primer** | **Region of amplicon sequences** | **Sequence (5’ — 3’)** |
| --- | --- | --- |
| PCR and PCDR primers | | |
| FP1 | ORF1b region (NC_045512:c.19492_19610)  https://www.ncbi.nlm.nih.gov/nuccore/NC_045512 | agacatcatgctaatgagtacag |
| RP1 |  | gtaaaagtgttccagaggttataag |
| FP2 |  | tcgatgcttataacatgatgatctcagc |
| RP2 |  | gtttgtaaacccacaagctaaagcca |
| LAMP and PCR-LAMP primers | | |
| F3 | ORF1ab region (NC_045512:c.12614-12808)  https://www.ncbi.nlm.nih.gov/nuccore/NC_045512 | tctgtcaaattacagaataatgag |
| B3 |  | atcactcttagggaatctagc |
| FIP |  | tcagtgcaagcagtttgtgtagtacttagtcctgttgcactacga |
| BIP |  | tgacaatgcgttagcttactacaaccatttcaaatcctgtaaatcgga |
| LF |  | agcagcacaagacatctgtcg |
| LB |  | cacaacaaagggaggtaggtttg |


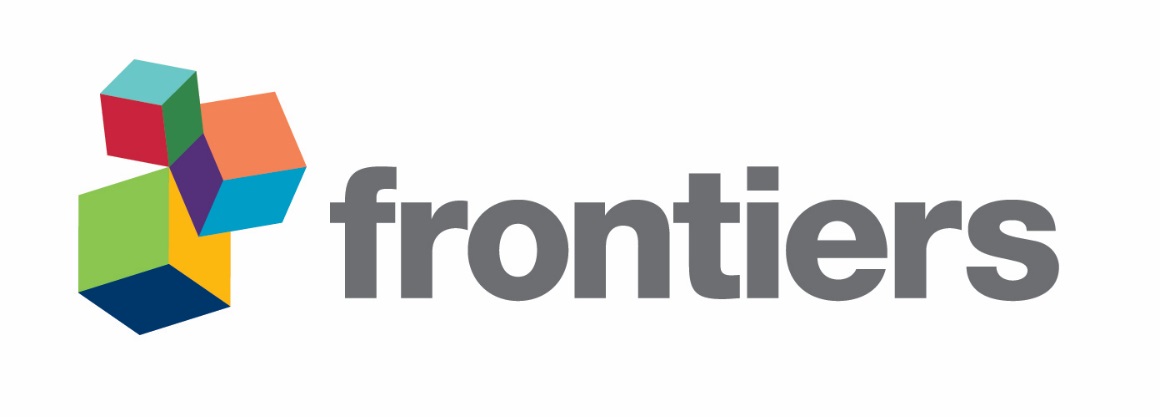

Supplement: Supplementary file 1 [file Table_1.DOCX]
